# Numerical Study of Customized Artificial Cornea Shape by Hydrogel Biomaterials on Imaging and Wavefront Aberration

**DOI:** 10.3390/polym13244372

**Published:** 2021-12-14

**Authors:** Yu-Chi Ma, Chang-Tsung Hsieh, Yu-Hsiang Lin, Chi-An Dai, Jia-Han Li

**Affiliations:** 1 Department of Engineering Science and Ocean Engineering, National Taiwan University, Taipei 10617, Taiwan; r09525048@ntu.edu.tw (Y.-C.M.); freddy50813@gmail.com (C.-T.H.); r08525055@ntu.edu.tw (Y.-H.L.); 2 Department of Chemical Engineering, National Taiwan University, Taipei 10617, Taiwan; polymer@ntu.edu.tw

**Keywords:** artificial cornea, hydrogel polymers, wavefront aberration

## Abstract

The blindness caused by cornea diseases has exacerbated many patients all over the world. The disadvantages of using donor corneas may cause challenges to recovering eye sight. Developing artificial corneas with biocompatibility may provide another option to recover blindness. The techniques of making individual artificial corneas that fit the biometric parameters for each person can be used to help these patients effectively. In this study, artificial corneas with different shapes (spherical, aspherical, and biconic shapes) are designed and they could be made by two different hydrogel polymers that form an interpenetrating polymer network for their excellent mechanical strength. Two designed cases for the artificial corneas are considered in the simulations: to optimize the artificial cornea for patients who still wear glasses and to assume that the patient does not wear glasses after transplanting with the optimized artificial cornea. The results show that the artificial corneas can efficiently decrease the imaging blur. Increasing asphericity of the current designed artificial corneas can be helpful for the imaging corrections. The differences in the optical performance of the optimized artificial corneas by using different materials are small. It is found that the optimized artificial cornea can reduce the high order aberrations for the second case.

## 1. Introduction

The human eye can be considered as a complex optical system that is composed of several critical components. Among these optical components, cornea is the thinnest organ, which is only 0.5 mm in thickness. Needless to say, cornea is important for eyesight since it is highly transparent and contributes about 80% of the total diopter [1,2]. Patients with injuries or diseases of the cornea that result in blindness may need transplant surgery to restore their vision [3,4]. However, due to a shortage of human cornea donors, it takes a long time for the matching between donor cornea and patient and some cases may have allograft rejection from the donor cornea, and the vision of these patients who needs the donated corneas are often hard to recover during the long waiting time. Thus, developing artificial corneas with biocompatible properties will be helpful for them. Several artificial corneas have been developed, such as Boston keratoprothesis and Alphacor [5,6,7]. However, the artificial corneas are still in the process of research and development. The long-term results of clinical experiments for implanting AlphaCor into human eyes show that the artificial cornea implantations have some risks and complications [8]. Furthermore, the different types of artificial corneas in recent year are still under the animal experimental testing or clinical status [9]. Different materials have also been developed for the artificial cornea. For making artificial corneas, including glass in very early research and medical grade polymer materials in recent years [7]. Among these polymer materials, hydrogels have been widely used for biomedical applications [10,11,12]. In addition to possessing high percentage of water content, these hydrogel biomaterials with interpenetrating polymer network structure (IPN) also have better mechanical properties to withstand constant external forces [13,14,15]. A recent numerical study shows that the patient-specific models are able to use as the cornea modelling for biomechanics in predicting the refractive surgery [16]. Ideally, with these characteristics, artificial cornea can be custom manufactured by using a 3D printing method to incorporate individual’s biometric parameters [17]. Many researchers focus on increasing the biocompatibility and enhancing the mechanical properties of the biomaterials that make artificial corneas. The optical design of artificial cornea with proper shape is also very important to improve the patient’s vision quality. Several methods have been developed to analyze the quality of human cornea in the ophthalmology research, such as optical coherence tomography [18,19,20,21] and Shack-Hartmann wavefront sensor [22,23]. The mathematical results from the Shack-Hartmann wavefront sensor measurements can be expanded by the Zernike polynomials [24,25] and used to describe the aberrations.

In this study, two kinds of IPN hydrogel biomaterials with optimized transparency are considered and their mechanical properties are like the artificial cornea materials, poloxamer 407 diacrylate (P407DA)/polyacrylic acid (PAA) and P407DA/poly (hydroxyethyl methacrylate) (PHEMA). P407DA is a triblock copolymer, which can be used to form the hydrogel network [26]. PAA is a biocompatible material, which can form an IPN hydrogel biomaterial used in human cornea research [27]. PHEMA is a polymer that has biocompatibility and it can be used in biomedical applications, such as artificial corneas or contact lens [28,29,30]. Three different shapes (spherical, aspherical, and biconic) of artificial cornea have been considered. In order to access the optical performance of the artificial cornea, a human eye model has also been established. Recently, several eye models have been developed and have their own advantages in different situations [31,32,33,34,35,36,37]. Thus, it is important to choose the suitable eye model. In this work, the eye model used in the simulation is based on James Polans’ wide-field eye model, which uses wavefront aberration data with a reverse building optimization technique to construct the eye model [37]. The aberrations, image on retina, and the Strehl ratio of point spread function (PSF) will be used to analyze the optical performance in the research. To understand how the different shape designs of the customized artificial corneas by hydrogel biomaterials can improve patient’s eye sight, it is assumed that the eye model of the patient has some defects to mimic their visual problems. Two different cases are considered in the simulations. For the first case, the shapes of the artificial corneas are designed by assuming that patient still wears glasses after transplanting a designed artificial cornea. For the second case, the shapes of the artificial corneas have been designed by assuming that the patient does not need to wear the glasses to recover their vision after transplantation.

## 2. Materials and Methods

In Section 2.1, the IPN hydrogel biomaterials used in the research are introduced. After that, the optical properties of the materials are described in Section 2.2. Furthermore, the mechanical properties of the IPN hydrogel biomaterials are also described in Section 2.3. To understand the optical performance of the artificial corneas, the parameters and properties of the established eye model used in the simulation are described in Section 2.4. In Section 2.5, three different shapes, biconic, aspherical, and spherical, and two different IPN hydrogel biomaterials, P407DA/PAA and P407DA/PHEMA, are used to design the artificial corneas. In Section 2.6, the image analysis methodology used in the research are described.

### 2.1. The IPN Hydrogel Biomaterials Preparation

**Figure 1:**
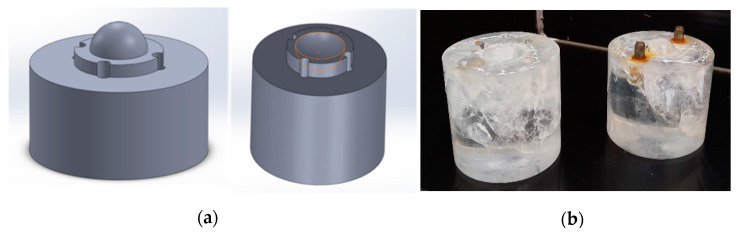
(**a**) Schematic diagram and (**b**) object of the artificial cornea mold.

In this research, two IPN hydrogel biomaterials, P407DA/PAA and P407DA/PHEMA, were chosen to design the shapes of artificial cornea. The P407 (Sigma, St. Louis, MO, USA), dehydrated tetrahydrofuran (THF) (Mallinckrodt, Staines, UK), trimethylamine (ACROS, Morris Plains, NJ, USA), acryloyl chloride (Alfa Aesar, Tewksbury, MA, USA), and excess n-hexane (Mallinckrodt, Staines, UK) were used to prepare the diacrylate-terminated poloxamer 407 (P407DA). To form IPN hydrogel biomaterials, P407DA was dissolved in deionized water. Then, the 2-hydroxy-2-methylpropiophenone (Sigma, St. Louis, MO, USA), which is the photoinitiator, was used and added a concentration of 1% by weight to the macromonomer. Then, the precursor solution was added into the mold as shown in Figure 1, and it was placed in the UV chamber (JH 1000-2, JIANN HAUR, Taoyuan, Taiwan) to polymerize. To prepare P407DA/PAA, the P407 hydrogels were immersed in the solution containing acrylic acid (AA) monomer, photoinitator, and dimethacrylate (TEGDMA) (Sigma, St. Louis, MO, USA) to crosslink material. Then, the UV source was used for the swollen P407 hydrogels in P407 preparation, and the P407DA/PAA IPN hydrogel biomaterial sample was obtained. A similar method was applied to prepare P407DA/PHEMA, and the solution of the P407 hydrogels were immersed in 2-hydroxyethyl methacrylate (HEMA) (Sigma, St. Louis, MO, USA), photoinitiator, and TEGDMA.

### 2.2. Optical Properties of the IPN Hydrogel Materials

**Figure 2:**
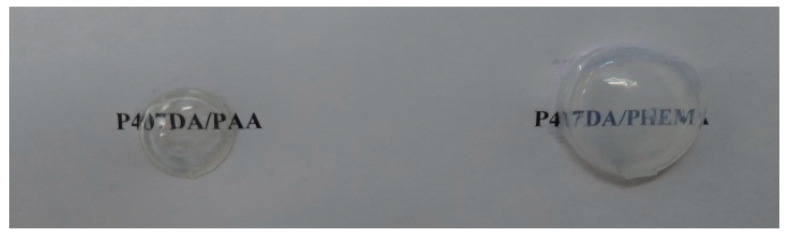
Samples made by P407DA/PAA and P407DA/PHEMA.

The optical properties of the IPN hydrogel biomaterials are shown in Table 1. A UV/VIS spectrophotometer (CARY 300, Agilent, Santa Clara, CA, USA) was used to detect the transmittance of the IPN hydrogel biomaterials in the wavelength range 380–780 nm. The average values of transmittances were shown in Table 1. A refractometer (NAR-3T, ATAGO, Bellevue, WA, USA) was used to measure the refractive index of the IPN hydrogel biomaterials, P407DA/PAA and P407DA/PHEMA, at the wavelength of 589 nm. The spectral sensitivities of the human cones had a peak in the wavelength range 500–600 nm [38]. Compared to human cornea, these IPN hydrogel biomaterials have high transmittances. The refractive index of these IPN hydrogel biomaterials is close to that of a typical human cornea at 589 nm. The hydrogel biomaterials, which are fabricated by using different processes, can have different transmittances, refractive indices. Figure 2 demonstrates the optical properties of the two IPN hydrogel biomaterials, P407DA/PAA and P407DA/PHEMA. These samples are made in a mold. The thicknesses of the central parts of these samples are close to 0.5 mm, which is similar to that of a typical human cornea. The refractive index of human cornea is approximately equals to 1.376 [39] and the transmittance is over 87% [37]. The results in Figure 2 shows good transparency, which corresponds to the measurement results in Table 1. These hydrogel samples also indicate that the material is robust in order to maintain the geometrical shapes of the artificial corneas shown in Figure 2.

### 2.3. Mechanical Properties of the IPN Hydrogel Biomaterials

The Young’s modulus was determined as absolute value of stress/strain ratio at 1% strain, and the water content of materials measured by drying method. The Young’s modulus represents the mechanical properties of the material which indicates that the material has high hardness when the Young’s modulus is a high value. In this situation, only few deformations occurred after the material has the stress. For the material of artificial cornea, it usually requires high mechanical strength to resist the intraocular pressure. For the P407DA/PAA, the Young’s modulus is 0.238 MPa and the water content is 73.45%. The Young’s modulus of P407DA/PHEMA is 0.9495 MPa and the water content is 49.4%. Compared with P407DA/PAA, the P407DA/PHEMA has higher mechanical strength, but the water content is low, which may affect oxygen permeability of the artificial cornea. 

### 2.4. Eye Model Construction in the Simulation

**Figure 3:**
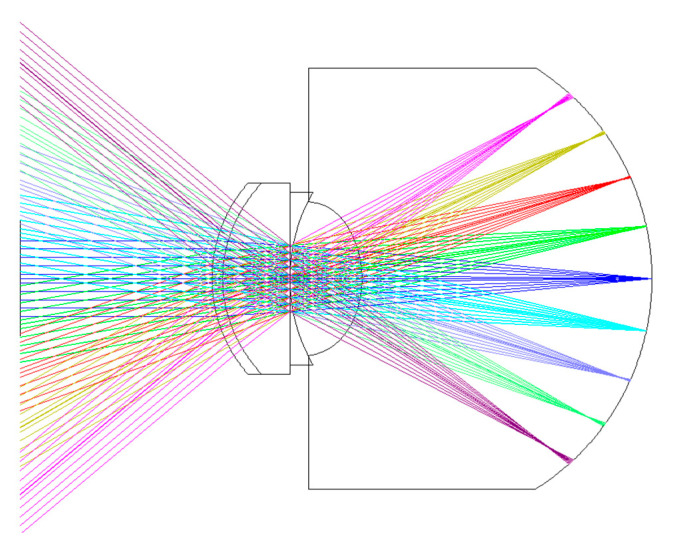
Structure of the eye model in the research, which refers to the J. Polan’s model.

For the light propagation from retina to cornea in the J. Polans eye model [37], which is in the adverse direction in this research, this eye model structure is referred to construct the wide eye model, which the propagation direction of light is from air, cornea to retina. The eye model structure in this research is shown in Figure 3. Table 2 shows the relevant parameters of Figure 3. This eye model has 4 mm of pupil size. The basic material properties of the cornea, crystalline lens, and vitreous humor are based on data from the research results of Atchison et al. [40] and Jaeken et al. [41]. In order to understand the image quality on the retina, the eye model in the simulation is constructed from cornea to retina. The gradient index of the crystalline lens in this eye model is based on the 30-year simplified schematic eye model of Goncharov and Dainty [42]. The gradient index of crystalline lens in the eye model is described as

(1) $$n=n_{0}+n_{r2}r^{2}+n_{r4}r^{4}+n_{r6}r^{6}+n_{z1}z+n_{z2}z^{2}+n_{z3}z^{3}+n_{z4}z^{4}$$

where $n_{0}$ is the refractive index in reference wavelength, $n_{r2}$, $n_{r4}$, $n_{r6}$, $n_{z1}$, $n_{z2}$, $n_{z3}$, and $n_{z4}$ are the coefficicents of the gradient index, *z* is the position along the optical axis, and *r* is the radius of curvature perpendicular to the *z* axis described as

(2) $$r^{2}=x^{2}+y^{2}$$

The decantation of the crystalline lens is ignored in the simulation. The biconic corneal surfaces are used to simulate the asymmetric property of the human cornea [43]. The biconic surface can be described as

(3) $$z=\frac{\frac{x^{2}}{R_{x}}+\frac{y^{2}}{R_{y}}}{1+\sqrt{1-\left(1+k_{x}\right)\frac{x^{2}}{R_{x}}-\left(1+k_{y}\right)\frac{y^{2}}{R_{y}}}}$$

where $R_{x}$ is the radius for transverse plane, $R_{y}$ is the radius for the sagittal plane, $k_{x}$ is the conic constant for the transverse plane, and $k_{y}$ is the conic constant for the sagittal plane.

### 2.5. Design of the Artificial Cornea

In this work, the human cornea material is replaced in the schematic eye model with the IPN hydrogel biomaterials. When the artificial corneas are designed, the designed shapes are needed to keep as anatomically correct as possible. The structure parameters of the shape of the artificial cornea include the corneal radius, corneal thickness, and the distance from the cornea to the crystalline lens. Hence, the weight of these factors were set equal to 1 × 10^6^ in the simulation process, which was large enough to reduce the effect from those factors and compare the difference between different type of the artificial cornea surface clearly. Based on the research results of Polans et al. [37] and Sun et al. [44], the designing parameters of current artificial cornea in the simulation are shown in Table 3. For the shape design, the radius, conic constant, anterior chamber thickness, and cornea thickness are considered in the design. It is found that neglecting the posterior cornea in simulation will lead to some errors in predicting the optical property of the human eye [45]. Therefore, the design parameters for the posterior cornea in this work are considered. Considering the difficulty of making the cornea model, three different shapes of the artificial corneas are designed, including biconic, aspherical, and spherical shapes. The biconic shapes can be described by Equation (3) as $R_{y}\neq R_{x}$ and $k_{y}\neq k_{x}$. The aspherical shape is a special case of biconic shape as $R_{y}=R_{x}$ and $k_{y}=k_{x}\neq 0$, and the spherical shape is the other special case of biconic shape as $R_{y}=R_{x}$ and $k_{y}=k_{x}=0$. The aspherical and spherical corneal surfaces can be described as

(4) $$z=\frac{cr^{2}}{1+\sqrt{1-\left(1+k\right)cr^{2}}}$$

where *c* is the curvature, *r* is the radius of curvature, and *k* is the aspherical coefficient.

### 2.6. The Diffractive Image Analysis Methodology of the Artificial Cornea

To have a clear central vision, the field of views in the simulation are set at +10, +5, 0, −5, −10 degrees along the meridian. Because the refractive index of the IPN hydrogel cornea was measured at 589 nm, the incident light wavelength is set as 589 nm in the simulation. In this study, the first 15 Zernike polynomials based on the standard notation from the Optical Society of America are considered for optimizing the artificial corneas. The Zernike polynomial can be described as Equations (5) and (6)

(5) $$Z_{n}^{m}\left(\rho ,\theta \right)=N_{n}^{m}R_{n}^{\left|m\right|}\left(\rho \right)\cos\left(m\theta \right);for\;m\geq 0$$

(6) $$Z_{n}^{m}\left(\rho ,\theta \right)=-N_{n}^{m}R_{n}^{\left|m\right|}\left(\rho \right)\cos\left(m\theta \right);for\;m<0$$

where *ρ* is the radial distance of unit circle, *θ* is the azimuth angle of unit circle, $N_{n}^{m}$ is the normalized value, and $R_{n}^{\left|m\right|}$ is the radial component, which can be described as

(7) $$R_{n}^{\left|m\right|}\left(\rho \right)=\overset{\frac{n-m}{2}}{\underset{k=0}{\sum }}\frac{{\left(-1\right)}^{k}\left(n-k\right)!}{k!\left(\frac{n+m}{2}-k\right)!\left(\frac{n-m}{2}-k\right)!}\rho^{n-2k};\;for\;n-m=even$$

(8) $$R_{n}^{\left|m\right|}\left(\rho \right)=0;\;for\;n-m=odd$$

The different value of *n* and *m* defined different aberration, where *n* = 1 or 2 is low-order aberration and *n* = 3 or *n* > 3 is high-order aberration. The piston, tip, and tilt are not considered here for the optimization because they are artifacts of the aberration measurement system [36]. The aberrations can therefore be analyzed from the calculated Zernike polynomials. The diffraction image analysis uses an optical transfer function (OTF) to calculate the image performance. The near focus is −1.4 D for the data of wavefront phase difference in initial human eyes model approximately. For analyzing the effect from different artificial cornea shapes in Section 2.4, the standard Zernike phase plate is added behind the lens in the model to correct the initial model phase difference to 0. For simulating diffraction images for different artificial cornea shapes, the standard Zernike phase plate is put in the same position and the image analysis is performed. The aberration and diffraction image analysis are used to judge the optical quality of the designed artificial corneas. In the simulation setting, a grayscale “F” letter is used and its letter size is set to 0.025 mm to conform with the 20/20 Snellen vision standard. To understand the quality of the image more intuitively, the Strehl ratio is also discussed in this paper. The Strehl ratio is a ratio of the PSF peak intensity of the aberration-free eye to the PSF peak intensity of the normal eye [46].

## 3. Results and Discussion

In this section, the simulation results of the eye model and the artificial corneas are discussed. The simulation model is based on the eye model in reference [37] to make sure of the accuracy and reliability of the simulation. In Section 3.1, the optical performance in two different situations are discussed. One situation is the eye model with glasses and the other situation is the eye model without glasses. In Section 3.2 and Section 3.3, the simulation results of the artificial corneas that use different IPN hydrogel materials and different geometrical shapes are presented. For the design of the artificial corneas, two cases have been considered in this research. In Section 3.2, the case discussed is of a patient that still wears glasses after being transplanted with an artificial cornea. In Section 3.3, the case discussed is a patient who recovers from a visual problem after transplanting with an artificial cornea. To know the optical performances of the artificial corneas, Zernike aberrations, image on retina, and the Strehl ratio are discussed in this research. Moreover, the biocompatibilities of different shapes are also the important issues for artificial corneas. Although the material biocompatibility experiments are not performed in this research, the effects of optical performances caused by various shapes are discussed in Section 3.2 and Section 3.3. Considering the tolerance of shapes, their corresponding Strehl ratios are simulated. 

### 3.1. Optical Performance of the Eye Model in the Simulation

The optical power of this eye model is found to be +61.45 D on-axis for the paraxial beam. The total length of this eye model is about 23.96 mm. It is assumed that the patient wears the glasses to correct the visual problem, and the glasses are designed for the patient. Table 4 shows the parameters of the glasses in the simulation. N-Bk7 is used as the material of the glasses. The glasses are designed as the concave lenses and constructed as an asymmetrical structure. 

**Figure 4:**
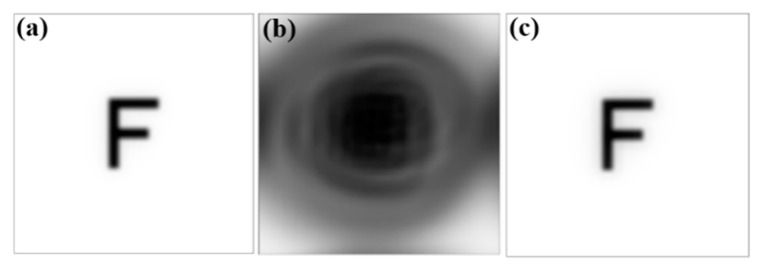
Images of (**a**) object, (**b**) simulated results on retina for the case without the glasses, and (**c**) simulated results on retina for the case with the glasses.

**Figure 5:**
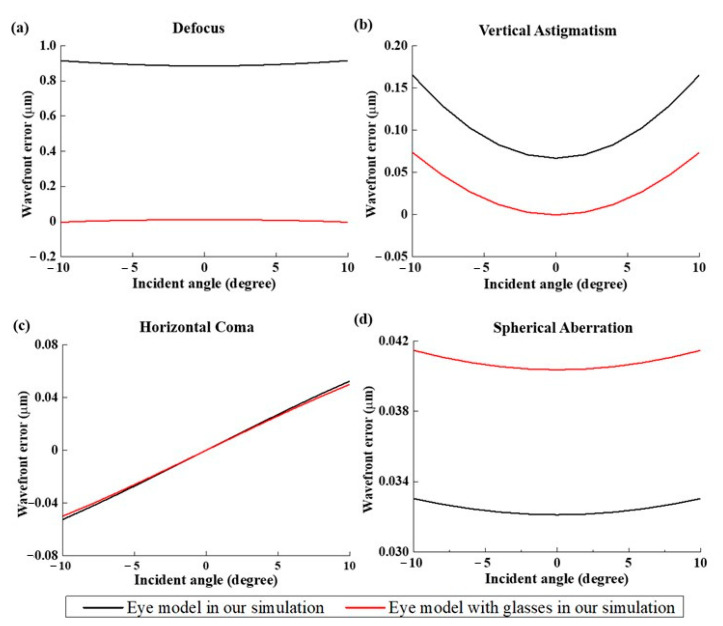
Plots of the Zernike aberration terms versus incident angle at 589 nm incident wavelength for transverse plan. Four most common optical aberrations are shown, including (**a**) defocus, (**b**) vertical astigmatism, (**c**) horizontal coma, and (**d**) spherical aberration.

Figure 4 show the image of the object, the images on retina with or without glasses. First, a letter “F” in the object plane is put in simulation, as shown in Figure 4a. Then, a collimated light enters into the eye model on the axis and forms an imaging on the retina. Two image results are discussed in the simulation. Figure 4b,c show the imaging results on the retina with or without the glasses, respectively. The result in Figure 4c shows that the image is much clearer than Figure 4b. This means that the vision can be corrected well after wearing the glasses. Figure 5 shows the results of the aberration contribution versus the incident angle relative to the transverse plane in the eye model without or with the glasses. In the case of eye model with glasses, according to the simulation analyzed by Zernike polynomial, the defocus and vertical astigmatism in the eye model with glass become lower than the original model. On the other hand, the horizontal coma is totally the same as the original model, and spherical aberration is more than the original model. The results in Figure 5 show the glasses can only be used to correct the low-order aberrations such as defocus and astigmatism, but it still cannot be used to correct the higher-order aberrations such as coma and spherical aberration. Almost 80% of the total loss of visual acuity is caused by the low-order aberrations [47] such that the image become clearer after placing the suitable glasses. Although under 20% of the total aberration is caused by the high order aberrations, it can still significantly affect the image quality [46]. In Figure 4c, the blurring at the border of the “F” letter can be found and it may be caused by the spherical aberration.

### 3.2. Optical Performance of the Eye Model with the Glasses after Replacing the Artificial Cornea

**Figure 6:**
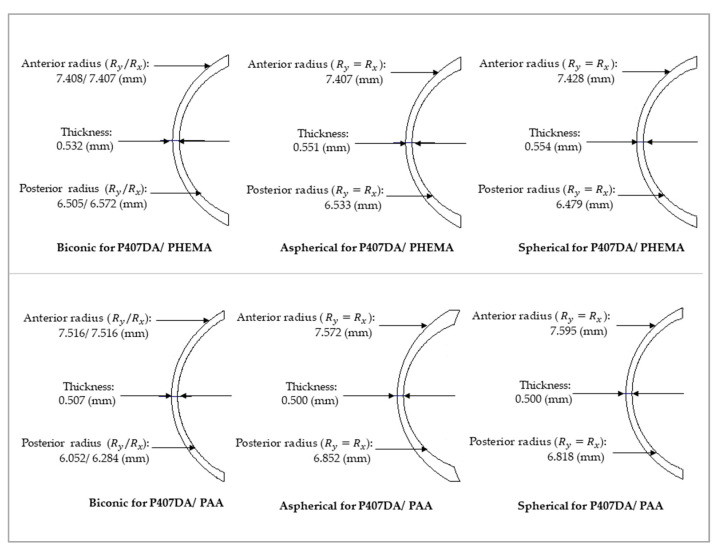
Various shapes of IPN hydrogel biomaterials in the cases with glasses.

**Figure 7:**
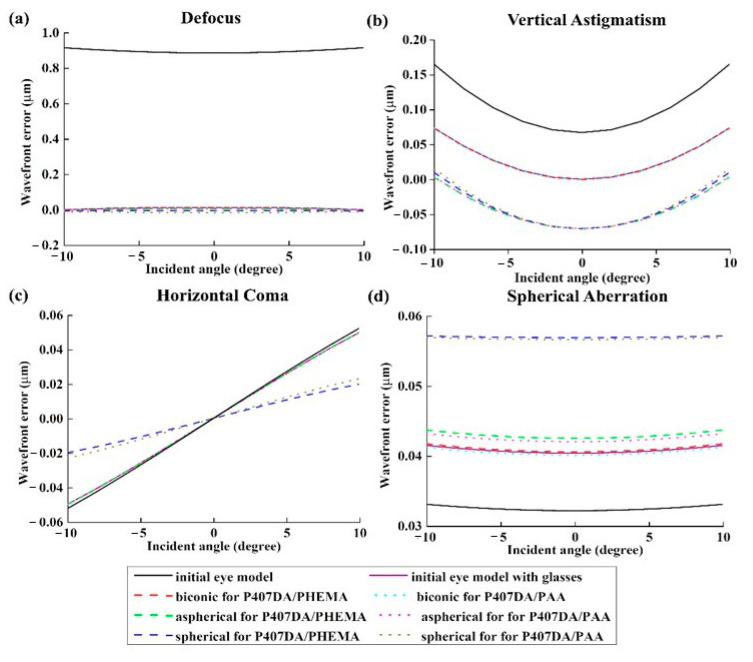
Plots of the Zernike aberration terms versus incident angle at 589 nm incident wavelength for transverse plane. This design aim of the artificial corneas is to let the optical performance of the artificial corneas similar to the initial eye model cornea. The black line and purple line in this figure are taken from Figure 3. Four optical aberrations are shown, including (**a**) defocus, (**b**) vertical astigmatism, (**c**) horizontal coma, and (**d**) spherical aberration.

**Figure 8:**
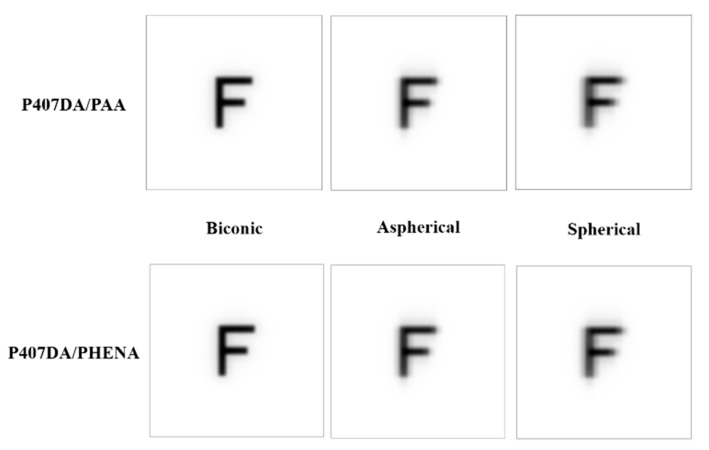
Image analysis of six different artificial corneas on the retina. In this case, the aim of the design is to replace the human cornea with the artificial cornea, and the optical performance of the artificial corneas is similar to the initial eye model cornea. The glasses are put in front of the models.

**Figure 9:**
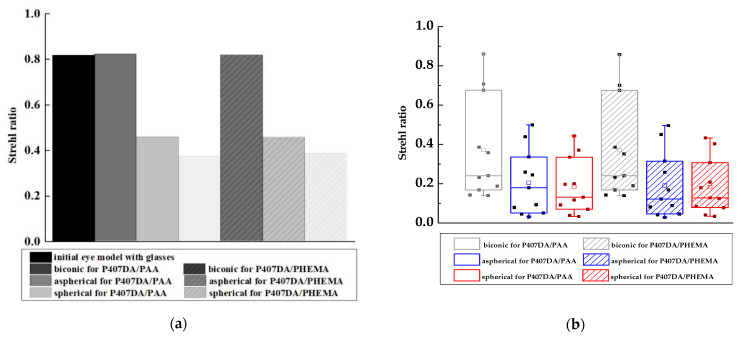
Strehl ratio of PSF of the artificial corneas and the initial eye model with glasses. In this case, the aim of the design is to replace the human cornea with the artificial cornea, and the optical performance of the artificial corneas is similar to that of the initial eye model cornea. (**a**) is the Strehl ratio of PSF without tolerance and (**b**) is the results that have considered the tolerances of the mold and the solid points on the diagram show the results in different tolerances.

It was assumed a patient still wears the glasses after transplanting with an artificial cornea in the case of this section. Thus, we use the glasses in Section 3.1 and optimize the design of the artificial corneas to let the optical performance of the artificial corneas be similar to the initial eye model cornea. The optimized target of the first 15 Zernike polynomials, except piston, tip, and tilt, are used to design the artificial cornea. The optimized weights of the Zernike polynomials are all setting as 10 from the incident angle +10 degrees to −10 degrees in this case. Figure 6 and Table 5 shows the parameters of the different artificial corneas. Figure 6 shows the designs of various shapes for biomaterials, which are considered in this research. The shape of human cornea is referred from the cornea parameters in the J. Polan’s model [37]. The thickness of the artificial corneas using the P407DA/PHEMA are closer to human cornea. Figure 7 shows the individual aberration terms of various artificial corneas in the transverse plane. In Figure 7a–c, the aberrations of initial eye model and model with glasses show that the defocus and vertical astigmatism are obviously corrected in the model with glasses, and the horizontal coma in the model with glasses is approximately the same as the initial eye model. However, the spherical aberration is increased in the model with glasses, as shown in Figure 7d. The results show that the model with glasses has smaller defocus and vertical astigmatism, but has more spherical aberration than the initial model. For the effects of different artificial cornea shape design, Figure 7a shows that the artificial corneas could efficiently decrease defocus greater than initial eye model. In Figure 7b, the results show that the artificial corneas could also decrease the vertical astigmatism, and the spherical and aspherical artificial corneas are more effective to reduce the vertical astigmatism. This is because the spherical and aspherical artificial corneas have a symmetrical optical structure. The eye model becomes an asymmetrical optical system for the cases when transplanting with the spherical and aspherical artificial corneas. After wearing glasses to reduce the astigmatism, the eye models with aspherical and spherical artificial corneas exhibit about −0.07 μm of vertical stigmatism error in this case. Furthermore, Figure 7c shows the horizontal coma has efficiently been reduced by spherical artificial cornea. However, the results in Figure 7d show that the horizontal coma has obvious addition in the artificial corneas and obviously increase in the spherical artificial corneas. The spherical artificial corneas cannot reduce the spherical aberration because its shape is without asphericity. After wearing the glasses, the results of spherical aberration for the eye model with the spherical corneas are also worse than the results for the initial eye model. It causes about 0.04 μm of a spherical aberration error in this case. Figure 8 shows the image analysis of six different artificial corneas on the retina. The results in Figure 7 also show that the materials in these cases have no much effect on correcting aberrations. Compared to two types of IPN hydrogel biomaterials that are used in the same shape design, they have nearly equal wavefront error and approximately the same trend in Figure 7. Consistent with the results shown in Figure 7, the results in Figure 8 show that the image for the case with biconic artificial cornea is clearer than the other cases. The image blurring found in the case of the aspherical artificial corneas may be caused by the astigmatism. The image blurring of the spherical artificial corneas is not only caused by the astigmatism, but also caused by the spherical aberration. In order to understand the image quality of the image more intuitively, Figure 9a shows the Strehl ratio of PSF of the artificial corneas to the initial eye model with glasses. The results show that the biconic artificial corneas are similar to the initial eye model with glasses. The Strehl ratio for the aspherical and spherical artificial corneas is low, which is a main reason causing the image blurring. Figure 9b shows the simulation results in consideration of the tolerance of the mold, and it is assumed that the tolerance range of the mold is ±1%. It is considered that the anterior radius of $R_{y}$ and the posterior radius of $R_{y}$ have same tolerance percentage in the same simulation case without thickness change. Furthermore, 0.2% tolerance percentage difference between simulation cases in same shape design. Table 6 shows the simulation results of the cases with 1%, ±0%, and −1% tolerances. Compared with the cases without tolerance in Figure 9a, the Strehl ratio in Figure 9b had been clearly changed with different tolerances. In Table 6, the Strehl ratio shows the noticeable differences in 1% tolerance and the optical performance is reduced when the tolerance change has occurred. The results show that the optical performances of artificial cornea would have been affected when the large tolerance occurred. To sum up the above results, the biconic shape is the best choice in the current system. Comparing to the material differences, it is found that with a proper design of the artificial corneas, the difference of the optical performances between two IPN hydrogel biomaterials is relatively small. The biggest difference occurs in the spherical artificial corneas. The wavefront error difference is about 0.015 μm in defocus. From the result of the retina images and the value of the Strehl ratio, there are few differences between the two different materials. To sum up, with a proper design, the differences between different materials is small in the current system. 

### 3.3. Optical Performance of the Eye Model without the Glasses after Replacing the Artificial Cornea

**Figure 10:**
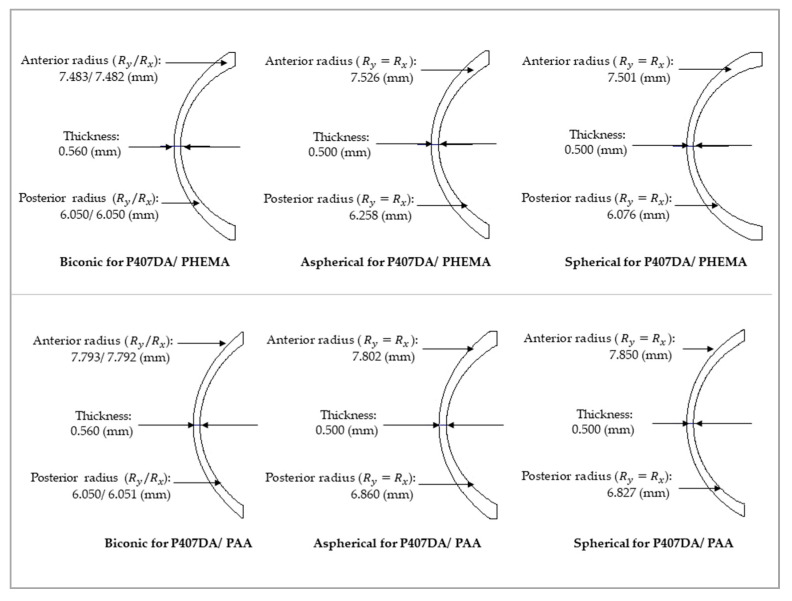
Various shapes of IPN hydrogel biomaterials in the cases without glasses.

**Figure 11:**
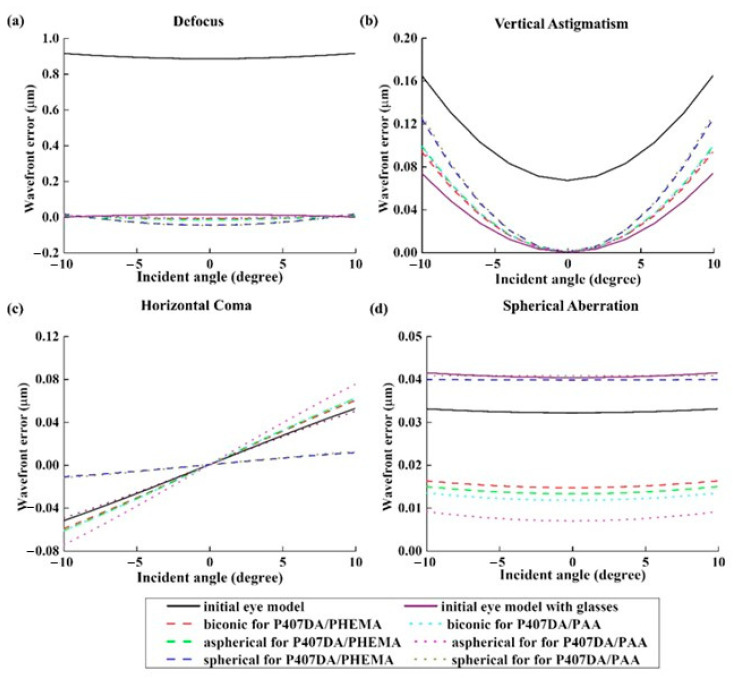
Plots of the Zernike aberration terms versus the incident angle at 589 nm for the transverse plane. In this case, the aim of the design of the artificial corneas is to reduce the aberration to recover the patient’s sight. The black line and purple line in this figure are taken from the results of Figure 6. Four optical aberrations are shown, including (**a**) defocus, (**b**) vertical astigmatism, (**c**) horizontal coma, and (**d**) spherical aberration.

**Figure 12:**
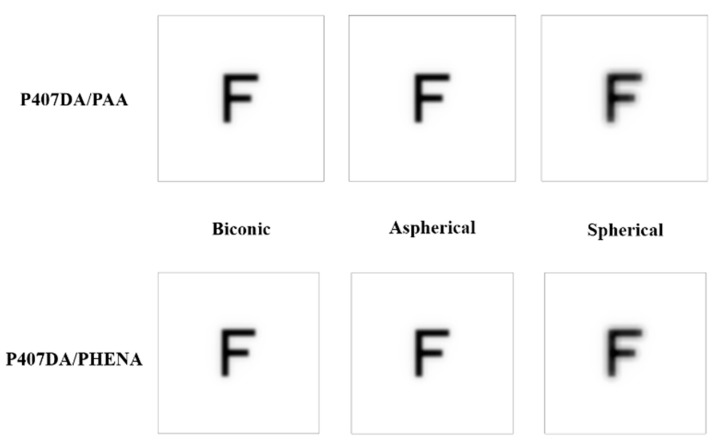
Image analysis of six different artificial corneas on the retina. In this case, the aim of the design of the artificial corneas is to reduce the aberration to recover the patient’s eye sight.

**Figure 13:**
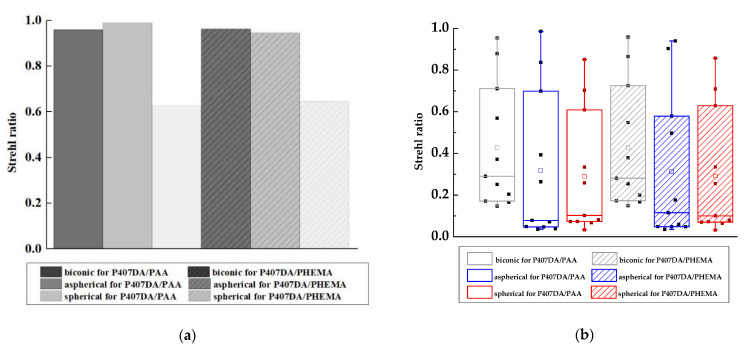
Strehl ratio of PSF of the artificial corneas and the initial eye model. In this case, the aim of the design is to reduce the aberrations to recover the patient’s eye sight. (**a**) shows the Strehl ratio without tolerance and (**b**) shows the results considering the tolerance of the mold in the cases without glasses.

In this section, it was assumed that a patient not only replaces with an artificial cornea, but also corrects the aberrations at the same time. It was assumed that the patient is not wearing the glasses to recover her/his visual problems. The design of the artificial corneas in this case was trying to reduce the aberrations of the initial eye model, as shown in Section 3.1. The optimized target of the first 15 Zernike polynomials, except piston, tip, and tilt, are set close to zero during the optimization for the artificial cornea. The weights of the optimization of the Zernike polynomials are all set to 10 from the incident angle of +10 degrees to −10 degrees. Figure 10 and Table 7 show the shape properties of artificial cornea. Compared with the shape designs in Section 3.2, the shape designs in this section are closer to the human cornea. Figure 11 shows the individual aberration terms for the various artificial corneas as a function of the incident angle relative to the transverse plane. In Figure 11a,b, the defocus and vertical astigmatism are efficiently reduced by the artificial corneas, and in Figure 11c, compared to different shape artificial corneas, the spherical artificial corneas can more effective correct the horizontal coma than other cases. Furthermore, the biconic and aspherical artificial corneas have more obvious horizontal coma than initial eye model. Additionally, in Figure 11d, the results show that the biconic and aspherical shapes have greater performance than spherical shape in spherical aberration correction. Moreover, similar to the results in Figure 7, the material choosing between two IPN hydrogel biomaterials in Figure 11 also has no much effect in the trend of aberration correction in same artificial cornea shape. The results in Figure 11 show that, with an appropriate design, the six different artificial corneas show an excellent correction ability for the aberrations. Not only for the low order aberration the defocus is decreased, but also for the high order aberration, the spherical aberration is decreased. During the optimization processes, it was found that it was hard to decrease the coma and spherical aberration at the same time in the simulations. It was a tradeoff between the coma and spherical aberration. Table 7 shows the structural and material parameters of the different artificial corneas used in the study. The results show that the values of the radius $R_{y}$, $R_{x}$ and conic $k_{y}$, $k_{x}$ of the biconic shapes are similar. As mentioned in Section 3.2, the eye model in the simulation is built by a symmetry structure except for the cornea. In order to correct the astigmatism, which is caused by asymmetric surface, the shape of the artificial cornea must be symmetrical. So, it causes the values of the radius $R_{y}$, $R_{x}$, and conic $k_{y}$, $k_{x}$ similar to each other in this situation. In other words, the results of the biconic artificial corneas will be similar to those for the aspherical artificial corneas. However, the results show that they still have some differences as shown in Table 7 and Figure 11. The reason for the difference between biconic shapes and aspherical shapes may be related to the process of the optimization. The function of the optimization can be described by the following equation,

(9) $$F^{2}=\frac{\sum_{i}W_{i}{\left(V_{i}-T_{i}\right)}^{2}}{\sum_{i}W_{i}}$$

where F is the optimization functional, $W_{i}$ is the weight of the *i*th operand, $V_{i}$ is the current value of the *i*th operand, and $T_{i}$ is the target of the *i*th operand. In the simulation, $V_{i}$ is the value of the aberrations in the eye model and $T_{i}$ is the target value of the aberrations. During the process of the structure optimization, the same weights of the optimization targets are set. In the simulations, the defocus and astigmatism have larger values as compared to the coma and spherical aberration. Therefore, the process of the optimization stops when the optimized artificial cornea can be used to correct the low order aberrations effectively, but only slightly for the high order aberrations. In addition, the shape of the surface greatly affects the high order aberrations. Thus, the different parameters of the optimized structures may have similar aberration correction result. Figure 12 shows the image on the retina of the six different artificial corneas. The results show that with a proper design of the artificial corneas, it can significantly reduce the image blur in this case. The results show that the image quality of the biconic artificial corneas is similar to the aspherical artificial corneas. The spherical artificial corneas still cause the image blurring. It may be caused by the spherical aberration. Figure 13a shows the Strehl ratio of the point spread function (PSF) for the artificial corneas. The results show that the biconic artificial corneas are similar in function to the aspherical artificial corneas. The Strehl ratio of the spherical artificial corneas is low to cause the image blur. To sum up, the biconic and aspherical shapes have better optical performance than that for the spherical artificial corneas. Figure 13b shows the simulation results considering the tolerance of the mold and Table 8 shows the simulation results of the cases with 1%, ±0%, and −1% tolerances. The simulation method is the same as Section 3.2, and the results in this section also show that the tolerances would cause the change of the Strehl ratio. This situation also shows that the optical performances had been reduced when tolerance occurred. Comparing to the material differences, it is found that, with a proper design, there is only a subtle difference between the optical performances of two biomaterials. In the results of the retina images and the Strehl ratio, there is no obvious difference between different materials. To sum up, with a proper design, the difference between different materials is small in this case.

Comparing with the case for wearing glasses from Section 3.2., it can be found that although wearing glasses can be used to correct the low-order aberrations such as defocus and astigmatism to make the vision clear, it still cannot correct the high-order aberrations, especially spherical aberration in the simulation. Uncorrected, the higher-order aberrations cause minor image blur in the simulation. However, in real life, high order aberration will reduce retinal image at pupil dilation moments [46] or night vision. A lot of research shows that they can achieve the purpose of correcting high-order aberrations by customizing the contact lenses [48,49,50]. In this work, the results show that this purpose could be achieved by using the proper designs of the artificial corneas.

## 4. Conclusions

The optical performance of the artificial corneas designed by using different shapes and novel IPN hydrogel biomaterials has been studied numerically in this research. Two kinds of IPN hydrogel biomaterials, P407DA/PAA and P407DA/PHEMA, and three kinds of outer cornea shapes, biconic, aspherical, and spherical geometries, have been considered to design the artificial corneas. Two different design cases for the optimized artificial corneas are demonstrated. For the first case, it is assumed that a patient wears glasses after replacing the artificial corneas. After the optimization, it is found that the biconic artificial cornea has better optical performance than the artificial corneas with other shapes. The reason is that a biconic surface has the advantage of the asymmetric surface. Thus, it will not cause excessive correction of astigmatism after wearing the glasses. Two biomaterials are considered in the optimization process. Despite different shapes and different hydrogels for the artificial corneas, their optical performance has only subtle differences. For the second case, it is assumed that the patient does not wear glasses after replacing the optimized artificial corneas. It is found that the patient’s eye sight can be recovered after replacing the optimized artificial corneas. It shows that the surfaces of the optimized biconic artificial corneas have similar radius $R_{y}$ and $R_{x}$ and similar conic $k_{y}$ and $k_{x}$, i.e., the optimized biconic shape is similar to the aspherical shape. The reason is that the eye model in this work is built by symmetrical components except the cornea. To correct the astigmatism, which is caused by an asymmetric surface, the shape of the artificial corneas must be symmetrical. With the characteristics of asphericity, biconic and aspherical artificial corneas show that they have better optical performance than that for spherical artificial corneas. Compared with the results in which the patient still wears glasses after replacing the artificial corneas, the biconic and aspherical artificial corneas can be used to correct the spherical aberrations, which usually cannot be amended easily with wearing the glasses only. The results of this study show that the vision of a patient can be repaired by using appropriately designed artificial corneas. The method described in this study can be also used for the structures with other materials for the eye model to customize a patient’s artificial cornea

## Figures and Tables

**Table 1:** Optical properties of the hydrogel biomaterials.

	Transmittance (%)	Refractive Index (at 589 nm)
P407DA	95.1	1.3531
PAA	97.21	1.5270
PHEMA	99.57	1.5119
P407DA/PAA	95.45	1.3574
P407DA/PHEMA	91.92	1.4232

**Table 2:** Parameters of the eye model in the research.

Surface	Radius (*R_y_*, *R_x_*) (mm)	Thickness (mm)	Conic (*k_y_*, *k_x_*)
Cornea	7.480, 7.517	0.500	−0.130, −0.136
Anterior chamber	6.287, 6.704	3.639	−0.005, −0.303
Pupil	Inf.	0	0
Crystalline lens	$9.533\;(R_{y}=R_{x}$)	3.690	$-3.203\;(k_{y}=k_{x}$)
Vitreous humor	$-5.736\;(R_{y}=R_{x}$)	16.000	$-0.278\;(k_{y}=k_{x}$)
Retina	$-13\;(R_{y}=R_{x}$)	-	$0\;(k_{y}=k_{x}$)

**Table 3:** Range and weight of the structure design of the artificial corneas.

Notation	Range	Weight
Radius (anterior)	7.00~8.16 (mm)	1 × 10^6^
Radius (posterior)	6.00~6.90 (mm)	1 × 10^6^
Conic constant (anterior)	−0.3~0	1
Conic constant (posterior)	−0.3~0	1
Cornea’s thickness	0.5~0.56 (mm)	1 × 10^6^
Anterior chamber thickness	2.91~3.65 (mm)	1 × 10^6^

**Table 4:** Parameters of the glasses in the simulation.

Surface	Radius (*R_y_*, *R_x_*) (mm)	Thickness (mm)	Conic (*k_y_*, *k_x_*)
Glasses anterior surface	−15.399, −15.936	0.600	$-2.007\;(k_{y}=k_{x}$)
Glasses posterior surface	−16.340, −17.026	5.000	$-2.096\;(k_{y}=k_{x}$)

**Table 5:** Artificial cornea properties for different IPN hydrogel biomaterials. The design is aimed to produce optical performance of the artificial corneas similar to the initial eye model cornea. Cornea A means anterior cornea and cornea P means posterior cornea.

	Surface	Notation	Radius (*R_y_*, *R_x_*) (mm)	Thickness (mm)	Conic (*k_y_*, *k_x_*)
P407DA/PHEMA	Biconic	Cornea A	7.408, 7.407	0.532	−0.112, −0.092
		Cornea P	6.505, 6.572	3.567	2.3 × 10^−4^, −0.033
	Aspherical	Cornea A	$7.407,\;(R_{y}=R_{x}$)	0.551	$-0.094,\;(k_{y}=k_{x}$)
		Cornea P	$6.533,\;(R_{y}=R_{x}$)	3.520	$-0.026,\;(k_{y}=k_{x}$)
	Spherical	Cornea A	$7.428,\;(R_{y}=R_{x}$)	0.554	$0,\;(k_{y}=k_{x}$)
		Cornea P	$6.479,\;(R_{y}=R_{x}$)	3.633	$0,\;(k_{y}=k_{x}$)
P407DA/PAA	Biconic	Cornea A	7.516, 7.516	0.507	−0.144, −0.130
		Cornea P	6.052, 6.284	3.646	−0.065, −0.300
	Aspherical	Cornea A	$7.572,\;(R_{y}=R_{x}$)	0.500	$-0.126,\;(k_{y}=k_{x}$)
		Cornea P	$6.852,\;(R_{y}=R_{x}$)	3.633	$-0.217,\;(k_{y}=k_{x}$)
	Spherical	Cornea A	$7.595,\;(R_{y}=R_{x}$)	0.500	$0,\;(k_{y}=k_{x}$)
		Cornea P	$6.818,\;(R_{y}=R_{x}$)	3.650	$0,\;(k_{y}=k_{x}$)

**Table 6:** Simulation results of the Strehl ratio that considered the tolerances of the mold in the cases of wearing glasses.

	Surface	Tolerance (%)	Anterior Radius (*R_y_*) (mm)	Posterior Radius (*R_y_*) (mm)	Strehl Ratio
P407DA/PHEMA	Biconic	+1	7.48208	6.57005	0.139
		±0	7.408	6.505	0.857
		−1	7.33392	6.43995	0.142
	Aspherical	+1	7.48107	6.59833	0.029
		±0	7.407	6.533	0.496
		−1	7.33293	6.46767	0.046
	Spherical	+1	7.50228	6.54379	0.034
		±0	7.428	6.479	0.403
		−1	7.35372	6.41421	0.084
P407DA/PAA	Biconic	+1	7.59116	6.11252	0.14
		±0	7.516	6.052	0.86
		−1	7.44084	5.99148	0.142
	Aspherical	+1	7.64772	6.92052	0.031
		±0	7.572	6.852	0.499
		−1	7.49628	6.78348	0.051
	Spherical	+1	7.67095	6.88618	0.033
		±0	7.595	6.818	0.371
		−1	7.51905	6.74982	0.092

**Table 7:** Artificial cornea properties for different IPN hydrogel biomaterials. In this case, the aim of the design of the artificial corneas is to reduce the aberration to recover the patient’s sight.

	Surface	Notation	Radius (*R_y_*, *R_x_*) (mm)	Thickness (mm)	Conic (*k_y_*, *k_x_*)
P407DA/PHEMA	Biconic	Cornea A	7.483, 7.482	0.560	−0.283, −0.285
		Cornea P	6.050, 6.050	3.650	−0.326, −0.325
	Aspherical	Cornea A	$7.526,\;(R_{y}=R_{x}$)	0.500	$-0.213,\;(k_{y}=k_{x}$)
		Cornea P	$6.258,\;(R_{y}=R_{x}$)	3.645	$-0.090,\;(k_{y}=k_{x}$)
	Spherical	Cornea A	$7.501,\;(R_{y}=R_{x}$)	0.500	$0,\;(k_{y}=k_{x}$)
		Cornea P	$6.076,\;(R_{y}=R_{x}$)	3.638	$0,\;(k_{y}=k_{x}$)
P407DA/PAA	Biconic	Cornea A	7.793, 7.792	0.560	−0.278, −0.283
		Cornea P	6.050, 6.051	3.650	−0.308, −0.309
	Aspherical	Cornea A	$7.802,\;(R_{y}=R_{x}$)	0.500	$-0.330,\;(k_{y}=k_{x}$)
		Cornea P	$6.860,\;(R_{y}=R_{x}$)	3.650	$-0.304,\;(k_{y}=k_{x}$)
	Spherical	Cornea A	$7.850,\;(R_{y}=R_{x}$)	0.500	$0,\;(k_{y}=k_{x}$)
		Cornea P	$6.827,\;(R_{y}=R_{x}$)	3.650	$0,\;(k_{y}=k_{x}$)

**Table 8:** Simulation results of the Strehl ratio that considered the tolerance of the mold in the cases without glasses.

	Surface	Tolerance (%)	Anterior Radius (*R_y_*) (mm)	Posterior Radius (*R_y_*) (mm)	Strehl Ratio
P407DA/PHEMA	Biconic	+1	7.55783	6.1105	0.149
		±0	7.483	6.05	0.958
		−1	7.40817	5.9895	0.168
	Aspherical	+1	7.60126	6.32058	0.048
		±0	7.526	6.258	0.94
		−1	7.45074	6.19542	0.048
	Spherical	+1	7.57601	6.13676	0.032
		±0	7.501	6.076	0.629
		−1	7.42599	6.01524	0.079
P407DA/PAA	Biconic	+1	7.87093	6.1105	0.147
		±0	7.793	6.05	0.954
		−1	7.71507	5.9895	0.166
	Aspherical	+1	7.88002	6.9286	0.047
		±0	7.802	6.86	0.986
		−1	7.72398	6.7914	0.049
	Spherical	+1	7.9285	6.89527	0.033
		±0	7.85	6.827	0.609
		−1	7.7715	6.75873	0.081

## Data Availability

This research did not report any data.
